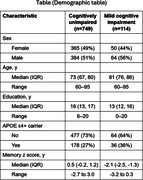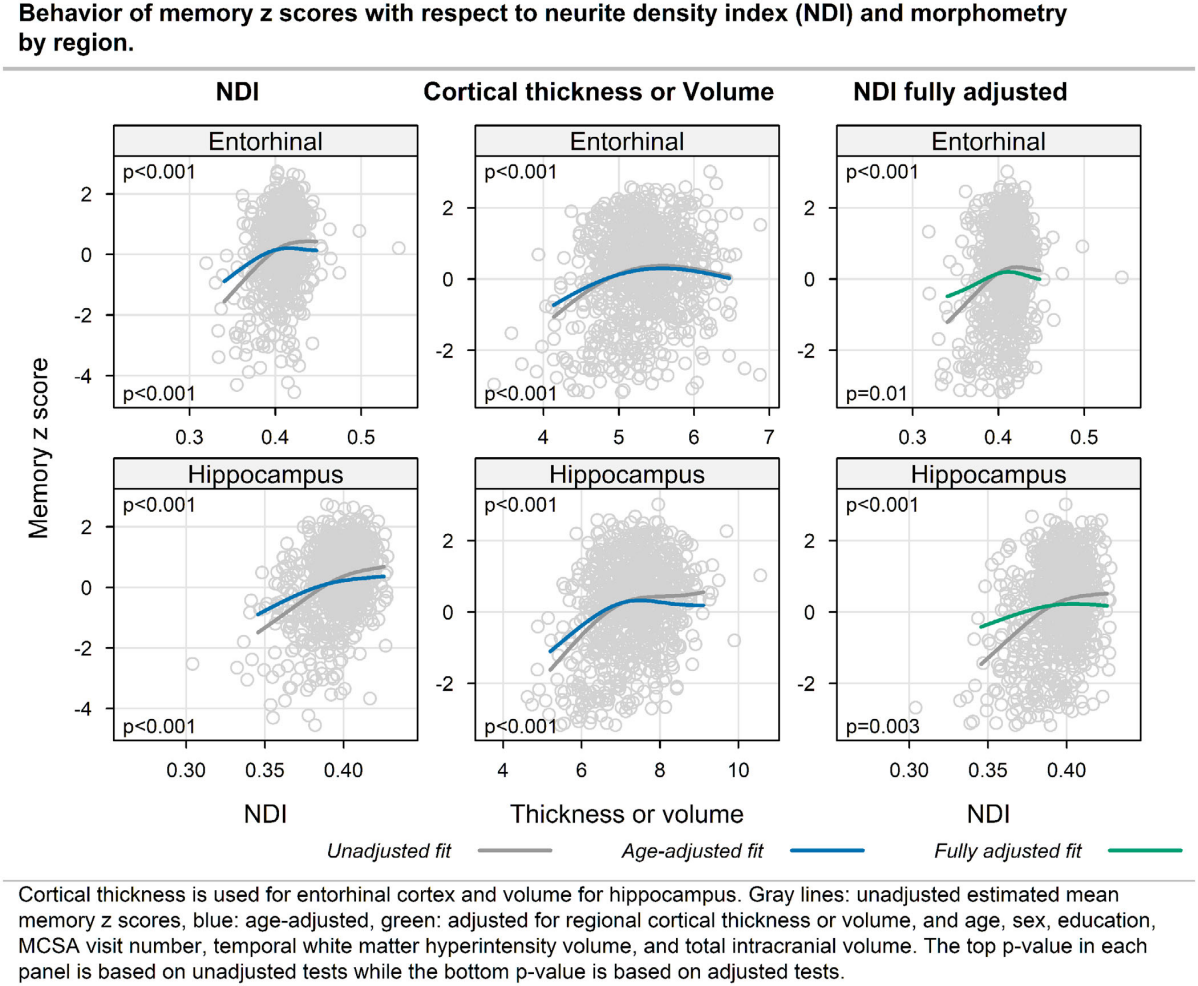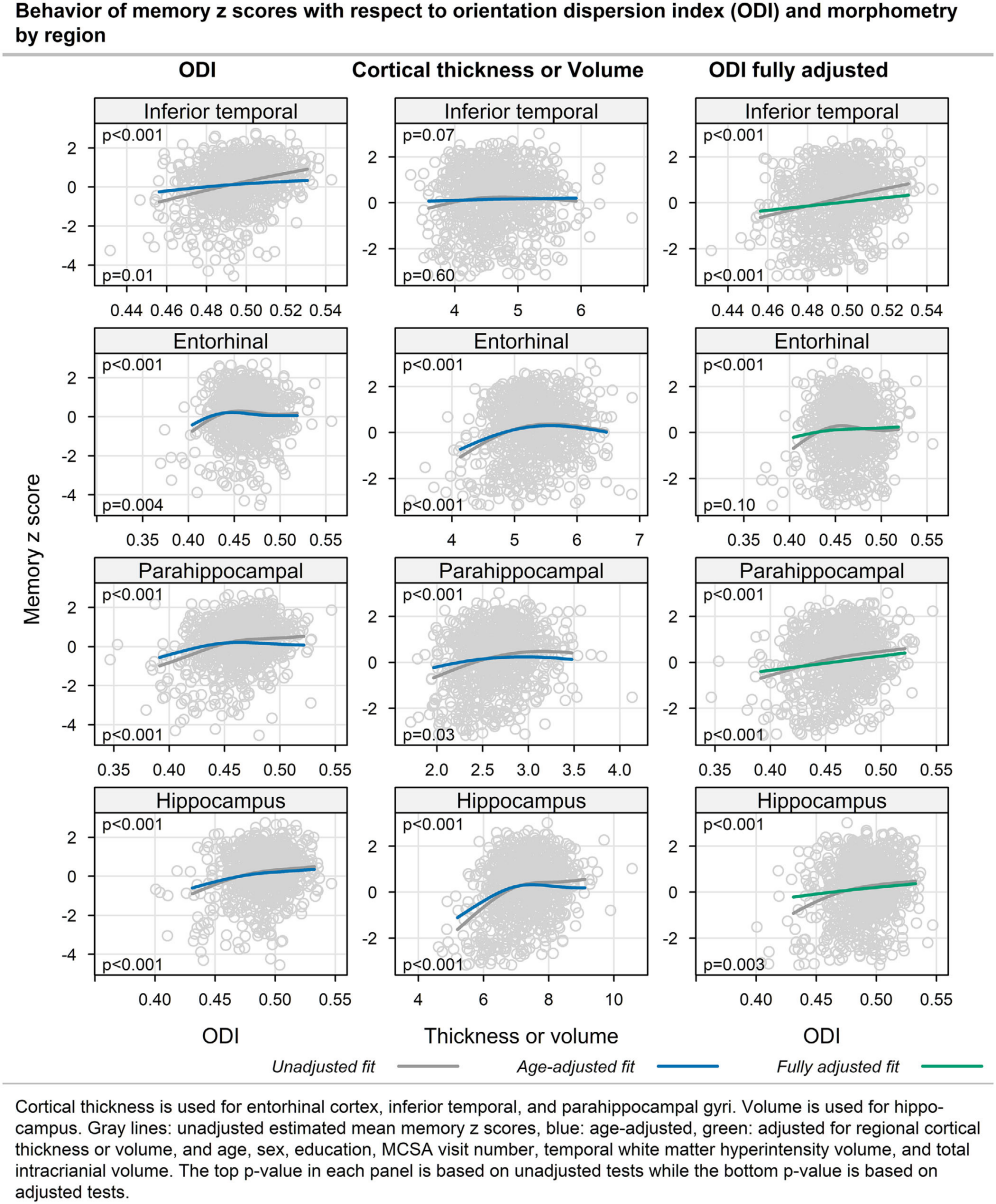# Association between temporal lobe cortical NODDI measures and memory function

**DOI:** 10.1002/alz.093997

**Published:** 2025-01-09

**Authors:** Jay J Pillai, Robert I. Reid, Stephen D. Weigand, Petrice M Cogswell, Prashanthi Vemuri, Mary M. Machulda, David S. Knopman, Jonathan Graff‐Radford, Ronald C. Petersen, Clifford R. Jack

**Affiliations:** ^1^ Mayo Clinic, Rochester, MN USA

## Abstract

**Background:**

Cortical structural changes in the temporal lobes including global and hippocampal volume loss and decrease in cortical thickness are well‐associated findings linked to cognitive decline in MCI and dementia patients. Neurite orientation dispersion and density imaging (NODDI) is a multishell diffusion MRI method enabling assessment of tissue microstructural integrity at the axonal and dendritic level. We wished to determine whether changes in temporal cortex orientation dispersion index (ODI) & neurite density index (NDI) correlate with decreases in memory function.

**Method:**

The MCALT_ADIR122 cortical atlas was used for ROI designation. NODDI imaging was obtained on a 3T Siemens Prisma scanner with 2 mm isotropic resolution. Participants (ages 60‐95 years) were cognitively unimpaired (CU) or had mild cognitive impairment (MCI). CU & MCI subjects were enrolled in the Mayo Clinic Study of Aging (MCSA) and underwent neuropsychological testing including 3 memory tests from which a composite memory z score was calculated. A generalized additive regression model was used to estimate z scores as a smooth function of ODI, NDI and cortical thickness or volume with adjustment for age, gender, education, MCSA cycle number, WMH and intracranial volume.

**Result:**

The Table displays demographic data. Figures 1 and 2 show the relationship of NDI and ODI measures to memory z scores across participants in individual ROIs both before and after adjustment for cortical thickness (“structural MRI”) as well as for confounds listed above. Both higher cortical thickness and higher NDI values in the hippocampus and entorhinal cortex were associated with significantly higher memory z‐scores (p<0.001) (Figure 1). Higher ODI values in addition to greater cortical thickness in temporal lobe regions, including within the hippocampus, parahippocampal gyrus, entorhinal cortex and inferior temporal gyrus, were associated with higher memory z‐scores (Figure 2). These significant associations (p <=0.01) were maintained after adjusting for cortical thickness in all these regions except entorhinal cortex for ODI.

**Conclusion:**

NODDI‐derived measures of microstructural integrity (NDI & ODI) within the temporal cortex may provide information about memory decline, beyond what cortical thickness analysis provides, and may be potentially useful for monitoring therapeutic effects of AD immunotherapy in the future.